# Malondialdehyde Serum Levels in Patients with Systemic Sclerosis Relate to Dyslipidemia and Low Ventricular Ejection Fraction

**DOI:** 10.3390/antiox12091668

**Published:** 2023-08-25

**Authors:** Zeina Ibrahim-Achi, Pablo Jorge-Pérez, Pedro Abreu-González, Raquel López-Mejías, Candelaria Martín-González, Miguel Á. González-Gay, Iván Ferraz-Amaro

**Affiliations:** 1 Division of Angiology and Vascular Surgery, Hospital Universitario de Canarias, 38320 Tenerife, Spain; zibrach@gobiernodecanarias.org; 2 Division of Cardiology, Hospital Universitario de Canarias, 38320 Tenerife, Spain; pjorpere@gobiernodecanarias.org; 3 Unit of Physiology, Department of Basic Medical Sciences, University of La Laguna, 38200 Tenerife, Spain; pabreu@ull.edu.es; 4 Epidemiology, Genetics and Atherosclerosis Research Group on Systemic Inflammatory Diseases, Instituto de Investigación Marqués de Valdecilla, 39011 Santander, Spain; rlopez@idival.org; 5 Department of Internal Medicine, University of La Laguna, 38200 Tenerife, Spain; mmartgon@ull.edu.es; 6 Division of Rheumatology, IIS-Fundación Jiménez Díaz, 28040 Madrid, Spain; 7 Division of Rheumatology, Hospital Universitario de Canarias, 38320 Tenerife, Spain

**Keywords:** systemic sclerosis, malondialdehyde

## Abstract

Systemic sclerosis (SSc) is a chronic disease characterized by vasculopathy with the involvement of dysfunctional microcirculatory vessels. Features of the disease include progressive fibrosis of the skin and internal organs and systemic inflammation characterized by the presence of circulating autoantibodies and proinflammatory cytokines. Furthermore, macrovascular disease and atherosclerosis are more common in patients with SSc than in the general population. Oxidative stress plays a crucial role in the development of several processes, including endothelial dysfunction, cancer, inflammation, and atherogenesis. Malondialdehyde (MDA) is a well-established marker of oxidative stress. In this work, we have analyzed the relationship between serum MDA levels and clinical, laboratory, and vascular characteristics in a well-characterized cohort of 53 patients with SSc. A multivariable analysis was performed to study the relationship between circulating MDA and disease characteristics in patients with SSc. Cardiovascular assessment was also performed, including ultrasonography of the carotid and aorta, and echocardiography. MDA showed a significant and positive relationship with the serum levels of lipid profile molecules such as total cholesterol (β coefficient = 0.006 (95% CI: 0.0004 to 0.01), nmol/mL, *p* = 0.037) and LDL cholesterol (β coefficient = 0.008 (95% CI: 0.001 to 0.01) nmol/mL, *p* = 0.017). On the contrary, most manifestations of the disease, including skin, lung, and joint involvement, as well as the presence of digital ulcers, were not related to MDA. However, high MDA levels were significantly and independently associated with lower ventricular ejection fraction after adjustment for covariates (β coefficient = −0.04 (95% CI: −0.06 to −0.02), nmol/mL, *p* = 0.001). In conclusion, serum MDA levels were related to higher levels of total and LDL cholesterol and a lower left ventricular ejection fraction in patients with SSc. MDA could serve as a potential biomarker of dyslipidemia and heart failure in SSc.

## 1. Introduction

Systemic sclerosis (SSc) is a chronic disease characterized by vasculopathy with the involvement of dysfunctional microcirculatory vessels, progressive fibrosis of the skin and internal organs and systemic inflammation characterized by the presence of circulating autoantibodies and proinflammatory cytokines [1]. Most patients with SSc can generally be classified based on the extent of skin involvement and the accompanying pattern of internal organ involvement, as well as the presence of overlapping features with other systemic rheumatic diseases. The major subsets of SSc include limited cutaneous SSc, diffuse cutaneous SSc, SSc sine scleroderma, and SSc overlap syndrome [2]. The overall incidence rates range globally from 8 to 56 new cases per million persons per year, and the prevalence rates fall between 38 and 341 cases per million persons [3]. Major clinical manifestations of SSc include skin thickening and hardening, Raynaud’s phenomenon and digital ulcers, arthritis, gastrointestinal involvement, interstitial lung disease, primary cardiac diseases like pulmonary artery hypertension, and kidney disease or myopathy [4]. Epidemiological studies indicate that survival in patients with SSc is reduced [5]. This is not only due to well-described prognostic factors such as skin extension and organ involvement, but also because the prevalence of cardiovascular disease, specifically macrovascular disease, in SSc patients is higher compared to healthy individuals [6,7,8]. In this sense, patients with SSc are at risk of developing atherosclerosis compared to people without this condition [9].

The pathogenesis of SSc is complex and remains incompletely understood. Nitric oxide, superoxide anions and oxidative stress imbalance are postulated to play a pathogenic role in SSc [10,11,12,13]. Protein phosphorylation, activation of various transcription factors, apoptosis, immunity, and cellular differentiation are all processes that rely on proper oxidation within biological systems. Mitochondria are the primary cellular source of reactive oxygen species (ROS) and are essential for aerobic metabolism and energy production through oxidative phosphorylation, which is facilitated by the respiratory chain [14]. Reactive oxygen species play important roles as second messengers in many intracellular signaling cascades aimed at maintaining the cell in homeostasis with its immediate environment [15]. Oxidative stress arises when the production of reactive oxygen species overwhelms the intrinsic antioxidant defenses. This leads to the oxidation of major cellular macromolecules such as DNA, lipids, and proteins, ultimately resulting in molecular dysfunction, necrosis, and apoptotic cell death [16]. Oxidative stress is believed to play a fundamental role in the development of various processes such as function of relaxation and proliferation of vascular smooth muscle cells, leukocytes adhesion, angiogenesis, platelets aggregation, thrombosis, vascular tone, and hemodynamics, among others [17]. Molecules that are modified by interactions with ROS in the microenvironment or that change in response to increased redox stress are biomarkers of oxidative stress. One of the most well-studied of these markers is malondialdehyde (MDA). In this sense, MDA is considered a byproduct of cellular peroxidation of polyunsaturated fatty acids and is widely recognized as a biomarker of oxidative stress and antioxidant status [18,19].

There is scarce information on the relationship of oxidative stress with the clinical characteristics of patients with SSc. A recent meta-analysis has confirmed that MDA, and other oxidative stress biomarkers, are upregulated in SSc [20]. However, no reports exist in a well-characterized series of SSc regarding the relation of MDA serum levels to a complete assessment of disease features. Therefore, in the present work, we have analyzed the relationship between serum MDA levels and clinical, laboratory, and vascular features in a well-characterized cohort of patients with SSc.

## 2. Materials and Methods

### 2.1. Study Participants

This is a cross-sectional study that included 53 patients with SSc. All were 18 years or older and met the American College of Rheumatology/European League Against Rheumatism 2013 classification criteria for SSc [21]. They had been diagnosed by rheumatologists and were periodically followed up at rheumatology outpatient clinics. For inclusion in the present study, SSc disease duration needed to be ≥1 year. None of the patients had established cardiovascular disease. Patients with a history of cancer or any other chronic disease, evidence of active infection, or a glomerular filtration rate <60 mL/min/1.73 m^2^ were excluded. The study protocol was ratified by the Institutional Review Committee at Hospital Universitario de Canarias and all subjects gave informed written consent (Approval code: EscleZ).

### 2.2. Assessments and Data Collection

Surveys were conducted on SSc patients to evaluate cardiovascular risk factors and medication usage. Participants completed a questionnaire and underwent a physical examination to measure anthropometric values and blood pressure levels. Medical records were carefully examined to identify specific diagnoses, medications, and any coexisting conditions. Hypertension was defined as having a systolic blood pressure exceeding 140 mmHg or a diastolic blood pressure exceeding 90 mmHg. The duration of SSc was determined by measuring the time elapsed since the first SSc-related symptom, excluding Raynaud’s phenomenon. SSc subtypes, namely limited and diffuse, were categorized based on the extent of skin thickening. The modified Rodnan Skin Score (mRSS) was used to assess skin thickening [22]. This score has frequently been employed as a measurable result in clinical trials. It assesses the intensity of these characteristics on a scale of 0 (typical) to 3 (highly severe) across 17 specific regions of the body and demonstrates an acceptable level of consistency within the same rater. Esophageal involvement was defined as the presence of any dysmotility indications observed through manometry. Articular involvement was identified by clinical signs such as joint swelling, deformities, contractures, and tendon friction rubs. Data on pulmonary function tests, forced vital capacity (FVC), forced expiratory volume (FEV), and diffusion capacity of the lung for the carbon monoxide (DLCO) were assessed. Interstitial lung disease was defined instrumentally by FVC  ≤  80%, FEV1/FVC  ≥  70% and/or DLCO  <  80% and interstitial changes on chest high-resolution computed tomography. Nailfold capillaroscopy was performed as previously described [23] using the CapillaryScope 200 MEDL4N microscope (Dino-Lite, Almere, The Netherlands). The assessment of nailfold capillaroscopy for all patients was conducted by a skilled operator to minimize potential bias. The procedure occurred at room temperature following a 20 min period of rest and encompassed both hands, examining all fingers except the thumbs. A comprehensive evaluation of the complete nailfold region was carried out using low magnification (50×). Reductions in capillary density, dilated capillaries, giant capillary structures, microhemorrhage, branching, disorganization, tortuosity, avascular area, and extravasation were investigated. Scleroderma patterns were subgraded as “early”, “active” and “late” [23].

Fasting serum samples were obtained and stored at −80 °C until the analysis of lipid levels in the bloodstream. Cholesterol, triglycerides, and HDL cholesterol were quantified using an enzymatic colorimetric assay (Roche Diagnostics, Basel, Switzerland). For the assessment of lipoprotein A and other lipoproteins, a quantitative immunoturbidimetric assay from Roche was employed. Cholesterol measurements fell within the range of 0.08 to 20.7 mmol/L, with an intra-assay coefficient of variation of 0.3%. Triglycerides exhibited a range of 4 to 1000 mg/dL, accompanied by an intra-assay coefficient of variation of 1.8%. HDL cholesterol values ranged from 3 to 120 mg/dL, with an intra-assay variation coefficient of 0.9%. The atherogenic index was calculated through the Castelli formula by using the ratio of total cholesterol to HDL cholesterol. LDL cholesterol was estimated using the Friedewald formula. High-sensitivity C-reactive protein (CRP) was measured using a standardized method. The assessment of insulin resistance (IR) utilized the homeostatic model assessment (HOMA) technique, which provided estimations of insulin sensitivity (%S) and β-cell function (%B) based on fasting plasma insulin, C peptide, and glucose concentrations. In this study, the updated HOMA2 computer model, known as HOMA2, was employed for these calculations [24].

The cardiovascular risk assessment tool, known as SCORE2, was formulated in line with the 2021 guidelines from the European Society of Cardiology concerning the prevention of cardiovascular diseases in clinical practice [25]. This scoring system categorizes risk into low to moderate, high, or very high, based on different age brackets (<50, 50–69, and ≥70 years). SCORE2’s primary purpose is to gauge the likelihood of experiencing fatal and non-fatal cardiovascular incidents over a span of 10 years, aimed at individuals aged between 40 and 69. Notably, for individuals aged 70 or above and in good health, the SCORE2-OP (Older People) algorithm has been developed. This alternative algorithm provides risk estimates for both 5- and 10-year periods, focusing on fatal and non-fatal cardiovascular events.

### 2.3. MDA Assessment

The Thiobarbituric Acid Reactive Substance (TBARS) assay was the method used to detect lipid oxidation. This assay specifically measures malondialdehyde (MDA), one of the end products generated during the breakdown of lipid peroxidation compounds. Serum levels of MDA were determined using a modified version of the method described by Kikugaw et al. [26]. To perform the assay, a 0.2 mL volume of the sample was combined with 0.2 mL of 0.2 M H_3_PO_4_ (Merck Life Science, Madrid, Spain). The color reaction was initiated by adding 25 µL of a 0.11 M thiobarbituric acid (TBA, Sigma-Aldrich, Madrid, Spain) solution. The mixture was then heated at 90 °C for 50 min using a heating block. After cooling, the TBARS (resulting in a pink complex color) was extracted by adding 0.4 mL of n-butanol (Sigma-Aldrich, Madrid, Spain). Centrifugation at 6000× *g* for 10 min allowed for the separation of the butanolic phase. Each sample was transferred to a 96-well plate and read at 535 nm using a microplate spectrophotometer reader (Spectra MAX-190, Molecular Devices, Sunnyvale, CA, USA). A calibration curve was prepared using authentic MDA standards (Merck Life Science, Madrid, Spain). The detection limit of the assay was determined to be 0.079 nmol/mL. The intra- and inter-assay coefficients of variation were calculated as 1.82% and 4.01%, respectively. The serum concentration of MDA was expressed in nmol per ml. To minimize potential interferences from compounds that react or absorb at 532 nm, each sample was accompanied by a blank tube (sample without the TBA reagent), and the absorbance of the blank tube was subtracted from each sample measurement [27]. In addition, the use of butanol as the stripping agent for the TBARS complex helped to mitigate many of these interferences [28].

### 2.4. Carotid, Aorta, and Echocardiogram Assessment

In patients with SSc, a carotid ultrasound examination was performed to evaluate the thickness of the carotid intima-media wall (cIMT) within the common carotid artery. The objective was to identify any localized plaques in the carotid arteries situated outside the skull (extracranial carotid tree). The measurements were carried out using the EsaoteMylab 70 ultrasound system from Genova, Italy. This system is equipped with a 7–12 MHz linear transducer and employs the Quality Intima Media Thickness in real-time (QIMT) automated software-guided radiofrequency technique developed by Esaote in Maastricht, Holland. The assessment process adhered to the guidelines established in the Mannheim consensus [29], which lays out criteria for identifying plaques within the accessible extracranial carotid arteries. These arteries include the common carotid artery, the bulb, and the internal carotid artery. Plaque criteria were established as the presence of a localized bulge within the arterial lumen, with a measurement of cIMT exceeding >1.5 mm. Additionally, the bulge needed to be at least 50% larger than the adjacent cIMT or result in an arterial lumen reduction of >0.5 mm [29]. Doppler ultrasound was used to evaluate blood flow patterns and velocities of left and right internal and common carotid arteries. The peak systolic velocity, the end-diastolic velocity, and the carotid ratio (the peak internal carotid artery velocity to common carotid artery velocity ratio) were assessed according to published guidelines [30]. A peak systolic velocity ≥ 125 cm/s or a ratio ≥ 2 are considered pathological thresholds.

A B-mode linear ultrasonography device (Siemens, Sunbury-on-Thames, UK) was employed to evaluate the dimensions of the aorta. The assessment process involved conducting a central longitudinal examination of the aorta, followed by acquiring transverse views at the renal level and at the point of the aorta’s greatest diameter. These steps were performed in alignment with the guidelines provided by the Society for Vascular Surgery [31].

Echocardiographic and Doppler studies were performed by an experienced cardiologist (P.J.-P.). In all cases, the cardiologist was blinded to clinical information. Transthoracic echocardiogram imaging was performed with all patients being in sinus rhythm. The calculation of the left ventricular ejection fraction was performed utilizing the biplane method of disk summation, which is a modification of Simpson’s rule. Meanwhile, the assessment of right ventricular systolic function involved measuring the Tricuspid Annular Plane Systolic Excursion (TAPSE), acquired from the tricuspid lateral annulus in the apical four-chamber view. These image acquisitions were conducted following the protocols outlined by the American Society of Echocardiography and the European Association of Cardiovascular Imaging [32]. The measurement of the anteroposterior diameter of the left atrium was conducted in the parasternal long-axis view, specifically at the conclusion of left ventricular systole. Evaluation of left ventricular diastolic function entailed capturing mitral diastolic filling velocities—the early diastolic filling peak (E) and the late diastolic filling velocity (A)—using pulsed Doppler for both mitral and aortic transvalvular flows. Additionally, pulmonary venous flow velocities were recorded. To acquire Doppler tissue images of the mitral annulus, a sample volume of 5 mm was positioned in the septal and lateral regions of the mitral ring through a four-chamber view. Further, an anterobasal and inferior location was selected using a two-chamber view. This allowed for the determination of early diastolic (e’) and end-diastolic (a’) velocities. Subsequently, the E/e’ ratio was calculated. For each patient, an average of three measurements was computed. The calculation of pulmonary artery systolic pressure was facilitated by analyzing the tricuspid regurgitation jet. This comprehensive approach adhered to the guidelines recommended by relevant bodies such as the American Society of Echocardiography [33].

### 2.5. Statistical Analysis

Demographic and clinical characteristics of individuals with SSc were presented using mean values (standard deviation—SD) or percentages when categorical variables. For continuous variables that did not adhere to a normal distribution, data were expressed as medians and interquartile ranges (IQR). To explore the association between disease-related data and MDA, a multivariable linear regression analysis was conducted. This analysis incorporated adjustments for confounding variables. Demographics and traditional cardiovascular risk factors that exhibited a relationship with MDA with a *p*-value below 0.20 were chosen as confounders. In other words, potential confounders were identified from demographic and traditional cardiovascular risk factors if their *p*-values in the univariable analysis were less than 0.20 regarding MDA. In the examination of the association between vascular parameters and MDA, certain lipid molecules derived from a formula (such as LDL cholesterol, LDL:HDL cholesterol ratio, non-HDL cholesterol, ApoB:ApoA1 ratio, and atherogenic index) were excluded due to collinearity. All analyses were carried out using Stata software, version 17/SE, developed by StataCorp in College Station, TX, USA. The statistical significance level was set at 5% for two-sided testing. Results with a *p*-value below 0.05 were considered statistically significant.

## 3. Results

### 3.1. Demographic, Laboratory, and Disease-Related Data

A total of 53 patients with SSc participated in this study, with 44 patients (83%) with the diffuse type and 9 patients (17%) with the limited type of disease. The mean age of recruitment was 60 ± 10 years. Table 1 shows the demographic characteristics of the patients and the characteristics related to the disease. Among the participants, 40% were hypertensive, 8% were current smokers, and 11% had been diagnosed with diabetes. Approximately 32% of the subjects were classified as obese, defined by a BMI of 30 kg/m^2^ or greater. The median SCORE2 in the population was 3.8 (interquartile range, IQR, 2.6–6.2), and the majority of patients (62%) fell into the low or moderate cardiovascular risk category. About a third of the patients were taking statins or aspirin as part of their treatment. Additional information on lipid profile and insulin resistance indices can be found in Table 1.

The levels of peripheral blood MDA measured in the study were 1.51 ± 0.72 nmol/mL. The average disease duration was 9 years (IQR 3–11, min–max 1–31). The mRSS score, a measure of skin involvement, was 4 (IQR 1–8). A subset of patients, about 19%, reported the presence of digital ulcers and calcinosis. At the time of the study, approximately 23% of the patients were taking prednisone with a median dose of 5 (IQR 5–7.5) mg/day, and 8% were taking methotrexate. Furthermore, 71% of the patients tested positive for anti-centromere antibodies, and 16% tested positive for anti-Scl70 antibodies. Other disease-related features are detailed in Table 1.

Table 2 shows the differences between patients with the limited and diffuse forms of SS. No differences were found in age, sex, and cardiovascular risk factors. Regarding lipid profile values, patients with the diffuse form showed significantly higher levels of HDL and apolipoprotein A1, and a lower atherogenic index. Regarding the characteristics of the disease, the differences found were consistent with the phenotype of each disease. Thus, anticentromere antibodies were more frequently found in the limited form, and antiScl70 in the diffuse one. The forced vital capacity was significantly lower in the diffuse form. No differences were found in the rest of the characteristics of the disease.

### 3.2. Relationship of Demographics and Disease-Related Data to MDA Serum Levels

The relationship between disease characteristics and MDA is shown in Table 3. Age and sex, body composition parameters and classic cardiovascular risk factors, as well as the SCORE2 calculator, did not show significant relationships with MDA levels. Only the use of aspirin disclosed a positive and significant association with higher levels of circulating MDA.

Indices of resistance to insulin action showed no relationship with MDA levels. However, several associations were found between lipid profile and MDA. In this sense, in the univariable analysis LDL cholesterol, non-HDL cholesterol, LDL:HDL cholesterol ratio, apolipoprotein B and atherogenic index showed a positive and significant association with MDA. A positive trend was also observed for total cholesterol and the ApoB:ApoA1 ratio (Table 3).

Regarding the characteristics of the disease, no relevant relationships were found between these and the levels of MDA. The presence of the diffuse type of the disease showed lower levels of MDA compared to the limited form. However, this difference was not statistically significant. The index of skin involvement, disease duration, capillaroscopy pattern, digital ulcers, pulmonary function tests, autoantibody pattern, or the use of different therapies did not show significant associations with MDA. Only the use of sildenafil (used in a single subject) showed a significant and positive relationship with MDA (Table 3).

The only variable that met the criteria to be considered as a confounding factor was the use of aspirin. Thus, lipid pattern and disease feature associations with MDA that disclosed a *p*-value less than 0.20 were adjusted for aspirin intake. Following this procedure, the results obtained were similar to those of the univariable analysis with minimal differences. In the lipid profile, the relationship between total cholesterol and MDA became significant, and that of the atherogenic index lost significance. Regarding the characteristics of the disease, the presence of gastric reflux was significantly related to lower values of MDA (Table 3).

### 3.3. Carotid, Aorta, and Heart Findings: Relationship with MDA

The mean peak systolic and end-diastolic carotid velocities were found to be within normal ranges not exceeding 125 cm/s. In addition, the ratio of maximum internal carotid artery velocity to common carotid artery velocity was less than 2 in all but one of the patients. Carotid plaques were found in 59% of the patients, and the diameter of the abdominal aorta was 1.5 ± 0.2 cm. Patients in the study exhibited a preserved left ventricular ejection fraction and tricuspid regurgitation maximum pressure gradient as shown in Table 4.

Data relating to the carotid and abdominal aorta did not reveal any significant association with MDA levels. Specifically, there was no correlation between the peak and end-diastolic velocities of the common and internal carotid arteries and the circulating levels of MDA. Similarly, there was no relationship between subclinical carotid atherosclerosis, assessed by the presence of carotid plaque or cIMT, and the circulating MDA levels. Furthermore, the diameter of the abdominal aorta also showed no significant association with MDA levels (Table 4).

Regarding the echocardiogram parameters, the lower left ventricular ejection fraction was associated with higher levels of MDA in the univariable analysis. This relationship was further analyzed by adjusting not only for aspirin intake but for lipid profile molecules, with a univariable relation to MDA of less than 0.20 (total cholesterol and apolipoprotein B). Due to collinearity, lipid molecules derived from a formula (LDL cholesterol, LDL:HDL cholesterol ratio, non-HDL- cholesterol, ApoB:ApoA1 ratio, and atherogenic index) were excluded from this linear regression model. Remarkably, the negative relationship between the left ventricular ejection fraction and MDA remained significant after this adjustment (Table 4).

## 4. Discussion

Our study demonstrates that the lipid profile of SSc patients correlates with serum MDA levels. Accordingly, MDA may play a role in the dyslipidemia of these patients, which could have a role in the accelerated cardiovascular disease of patients with SSc.

A meta-analysis of 47 studies published up to the end of 2015 showed elevated oxidative stress biomarkers in the plasma of SSc patients compared with healthy controls [20]. They included elevated levels of MDA, lipid hydroperoxides, nitric oxide, and asymmetric dimethylarginine, whereas the concentrations of superoxide dismutase and vitamin C were lower than in the control group. Fourteen articles included in this review evaluated plasma/serum MDA levels. The results of a random effects model showed that the level of MDA in patients with SSc was higher than in the control group (standardized mean difference = 0.51 (95% CI: 0.12 to 0.91), *p* = 0.011). However, these studies did not perform a complete characterization of patients with SSc and did not analyze a complete lipid profile, the association of MDA with aortic or carotid vascular studies, or echocardiography findings in patients with SSc.

The association of the lipid profile of SSc patients with serum MDA deserves further discussion as SSc has been associated with an altered lipid profile. Lower levels of HDL cholesterol and total cholesterol have been described in patients with limited cutaneous SSc compared with healthy individuals [34]. In another study that included patients with limited and diffuse SSc compared with controls, reduced serum HDL cholesterol levels, higher total cholesterol levels, and a higher atherogenic index were reported [35]. Furthermore, higher levels of lipoprotein A have been found in SSc patients compared to controls [36]. Similarly, cholesterol efflux capacity [37] and proprotein convertase subtilisin/kexin type 9 [38] have been reported to be downregulated in SSc patients compared to controls. Interestingly, a decrease in HDL was correlated with anti-centromere antibodies and the presence of pulmonary artery hypertension but not with the ESR or CRP level. On the contrary, another study including both limited SSc and diffuse SSc patients found lipid profile aberrations (increased LDL and triglycerides, decreased HDL, and no difference in total cholesterol concentrations) correlating with anti-topoisomerase I antibodies [39]. Current knowledge related to lipid alterations in SSc has been recently reviewed [40]. In our study, we found differences in some lipid molecules between patients with limited and diffuse forms. However, since nine patients had the diffuse form, these differences have to be considered with caution. We are not aware of studies that have compared the lipid profile between both types of SS. Despite these interesting observations, the underlying mechanisms responsible for these lipid profile changes and their relevance to the development of atherosclerosis in SSc patients remain unresolved. Given that MDA is one of the final products of the peroxidation of polyunsaturated fatty acids in cells, we believe that MDA levels, as an expression of the oxidative stress of the disease, could be involved, as a cause or consequence, in the lipid profile alteration found in SSc. It should be noted that the association described in our work was predominant in those molecules whose elevation is considered deleterious in terms of cardiovascular disease (total cholesterol, LDL cholesterol, apolipoprotein B, and atherogenic index).

Results on the relationship of MDA with the clinical characteristics of SSc have not been consistent. The presence of anti-Scl-70 was associated with higher levels of MDA in a study that included 28 patients [41]. In another report focused on the role of oxidative stress in the development of interstitial lung disease in patients with SSc, there was no correlation between plasma MDA levels and duration of disease, duration of pulmonary signs, values of pulmonary function tests, and high-resolution computed tomography scores [42]. In contrast, a study of 15 patients with SSc showed an inverse relationship between elevated plasma MDA levels and disease duration, but not with other disease characteristics [43]. Several therapies have been described to modify MDA levels. In this regard, MDA has been found to decrease after iloprost infusion [44] and the use of statins [45] or dihydropyridine calcium channel antagonists [46]. Remarkably, children with SSc had MDA levels similar to the control group [47]. Our study, which included a larger series of well-characterized patients, allowing us to adjust for covariates, showed that most clinical features of the disease do not appear to be related to this oxidative stress biomarker. This would imply that the organ manifestations of the disease, or its clinical phenotype, are not mediated by MDA, but MDA does have some pathophysiological role in particular characteristics like lipid pattern and endothelial damage. However, more studies are needed on this matter to answer this question.

The expression of MDA in other autoimmune diseases has been studied before. For example, in a recent report of our group of 284 patients with systemic lupus erythematosus (in press, https://doi.org/10.3390/antiox12081535, accesed on 15 April 2023), cumulative musculoskeletal and skin damage was associated with superior serum levels of MDA. In addition, activation of the complement system was also related to higher circulating MDA levels. In a study involving 80 patients with systemic lupus erythematosus and 80 healthy individuals as controls, researchers observed higher levels of circulating MDA in systemic lupus erythematosus patients [48]. These elevated MDA levels were also found to correlate with systemic lupus erythematosus disease activity score. Furthermore, patients with SLE’s neuropsychiatric manifestations, vasculitis, and anti-DNA antibodies exhibited higher MDA levels. However, when comparing MDA levels between patients with and without disease damage, no significant differences were detected [48]. Similarly, in a separate investigation consisting of 40 systemic lupus erythematosus patients and 50 controls, researchers also noted a significant increase in MDA levels [49]. Moreover, in a report on rheumatoid arthritis and systemic lupus erythematosus, levels of MDA were remarkably altered in RA and SLE patients compared to controls. Markers of increased oxidative stress and impaired antioxidant capacity were profound in rheumatoid arthritis and significantly reflected disease activity in both diseases, with special attention to alopecia and lupus nephritis. Rheumatoid arthritis patients receiving methotrexate had significantly altered parameters and the steroid dose in systemic lupus erythematosus patients correlated with these markers [50]. The fact that MDA is increased in these two diseases would support the idea that autoimmune diseases, like SSc, express dysfunctional values of this molecule and that, therefore, inflammation and autoimmunity would play a role in the abnormal expression of MDA.

In our study, patients with peripheral arterial disease or those who had had a previous cardiovascular event were not included. This exclusion criterion could have been a reason for not seeing a potential association of MDA with morphological damage of great vessels in SSc. In this sense, although almost sixty percent of the patients presented carotid plaque, the maximum systolic velocity was normal, indicating the absence of carotid stenosis. Furthermore, the abdominal aorta and echocardiography data were within normal ranges with, respectively, an absence of abdominal aortic dilatation and pulmonary hypertension. These results speak against large vessel disease in our SSc patients without a clinical history of cardiovascular disease. However, it is known that cardiovascular disease in SSc is generally silent and is generally unrecognized until late in the course of the disease [6]. Therefore, it cannot be ruled out that the pathological mechanisms are already underway despite the apparent normality detected in our study. In this regard, we observed a negative relationship between MDA and ventricular ejection fraction in patients with SSc. These observations are in agreement with data from a recent report on 774 patients with chronic heart failure with reduced ejection fraction. In these patients, MDA levels were found to be independent predictors of death and poor prognosis at a 1-year follow-up [51].

Other oxidative stress markers have been studied in SSc like F2-isoprostane, 8- hydroxy2deoxyguanosine or advanced oxidation protein products (AOPP) [52,53,54,55]. For example, F2-isoprostane (a product of ROS-mediated arachidonic acid peroxidation) and 8- hydroxy2deoxyguanosine (produced by the non-enzymatic random oxidation of tissue phospholipids by oxygen-derived radicals) have been shown to be higher in SSc patients than in controls [53]. 8-hydroxy-2′deoxyguanosine levels were significantly associated with the presence of pulmonary fibrosis, decreased forced vital capacity, and decreased alveolar volume, suggesting a potential predictive value of this biomarker [54]. Similarly, advanced oxidation protein products (AOPP) isolated from SSc patients have been found to stimulate endothelial cells and fibroblasts from healthy donors to generate ROS involved in vascular or fibrotic complications [54]. In a recent report, oxidative stress response in the peripheral blood was assessed in 55 patients with SSc and 44 well-matched controls using real-time monitoring of protein hydroperoxide formation by the coumarin boronic acid assay [55]. SSc was characterized by a significantly faster (2-fold) fluorescent product generation in the coumarin boronic acid assay and higher cumulative protein hydroperoxide formation (3-fold) compared to controls. The dynamics of protein hydroperoxide generation were not associated with the form of the disease (diffuse vs. limited SSc), current immunosuppressive therapy use, presence of abnormal nailfold capillaries, and autoantibody profile [55].

We acknowledge that we do not recruit controls. However, as we have discussed above, there is enough information to show that MDA levels are elevated in SSc compared to controls. Furthermore, our purpose was to study the expression of MDA in the clinical features of a well-characterized cohort of patients with SSc. The MDA reference range is 1.3–1.4 nmol/L [56,57] in healthy populations. Therefore, we believe that the values found in our patients (1.51 nmol/mL) support the previously mentioned notion that MDA is elevated in this disease. Another limitation of our study is due to its cross-sectional nature, which did not allow us to infer causality. We did not collect information regarding other antinuclear antibodies because we focused only on those that are related to the disease itself like anti-Scl-70 or anticentromere autoantibodies. For this reason, we cannot conclude how other disease autoantibodies are related to MDA in SSc. We also admit that SSc includes two different clinical phenotypes—limited and diffuse. This would represent that MDA may be expressed differently in each type. However, MDA did not differ between both types nor did both populations display important differences in comorbidities, analytical profile, and therapies used.

## 5. Conclusions

In conclusion, our work provides new data on the role of MDA as a marker of oxidative stress in patients with SSc. Serum levels of MDA were associated with higher levels of total and LDL cholesterol, supporting, therefore, the role of MDA in a harmful lipid pattern in this disease. In addition, there was a negative relationship between the left ventricular ejection fraction of these patients and the serum levels of MDA. We believe that circulating MDA could serve as a potential biomarker of dyslipidemia and heart failure in SSc patients. Further studies are needed in the future in order to evaluate the benefits of assessing MDA in these patients.

## Figures and Tables

**Table 1:** Demographics of systemic sclerosis patients.

	SSc Patients (n = 53)
Demographics	
Female, n (%)	49 (92)
Age, years	60 ± 10
BMI, kg/m^2^	29 ± 6
Waist circumference, cm	98 ± 14
Hip circumference, cm	105 ± 11
Waist to hip ratio	0.93 ± 0.07
Cardiovascular comorbidity	
Hypertension, n (%)	21 (40)
Current smoking, n (%)	4 (8)
Diabetes, n (%)	6 (11)
BMI > 30 kg/m^2^, n (%)	17 (32)
Statins, n (%)	19 (36)
Aspirin, n (%)	17 (32)
SCORE2 calculator	
SCORE 2, %	3.8 (2.6–6.2)
SCORE2 categories, n (%)	
Low to moderate	33 (62)
High	16 (30)
Very high	4 (8)
Laboratory data	
Malondialdehyde, nmol/mL	1.51 ± 0.72
CRP, mg/dL	2.0 (0.6–4.8)
Cholesterol, mg/dL	209 ± 33
Triglycerides, mg/dL	203 ± 90
HDL cholesterol, mg/dL	53 ± 13
LDL cholesterol, mg/dL	116 ± 30
LDL:HDL cholesterol ratio	2.29 ± 0.78
Non-HDL cholesterol, mg/dL	156 ± 31
Lipoprotein A, mg/dL	34 (13–85)
Apolipoprotein A1, mg/dL	163 ± 27
Apolipoprotein B, mg/dL	108 ± 25
Apo B:Apo A ratio	0.68 ± 0.21
Atherogenic index	4.2 ± 1.2
Insulin resistance indices	
Glucose, mg/dL	100 ± 22
Insulin, µU/mL	12.4 (6.1–19.3)
C-peptide, ng/mL	5.0 ± 3.2
HOMA2-IR	1.62 (0.85–2.51)
HOMA2-S%	62 (40–118)
HOMA2-B%-C-peptide	201 ± 106
Systemic sclerosis related data	
SS type, n (%)	
Limited	44 (83)
Diffuse	9 (17)
Disease duration, years	9 (3–11)
Modified Rodnan Skin Score, units	4 (1–8)
Raynaud phenomenon, n (%)	48 (91)
Digital ulcers, n (%)	10 (19)
Calcinosis, n (%)	10 (19)
Arthritis, n (%)	3 (6)
Gastric reflux, n (%)	30 (57)
Pathological esophageal manometry, n (%)	7 (50)
Nailfold capillaroscopy pattern	
Normal	9 (19)
Early	9 (19)
Active	15 (31)
Late	2 (4)
Unclassified or not valuable	13 (27)
Interstitial lung disease, n (%)	7 (14)
FVC, %	96 ± 16
FEV1, %	99 ± 18
DLCO, %	83 ± 16
Pulmonary hypertension, n (%)	2 (5)
Autoantibodies	
Anti-centromere antibody positivity, n (%)	35 (71)
Anti-Scl70 antibody, n (%)	8 (16)
Therapies	
Current NSAIDs, n (%)	9 (17)
Current prednisone, n (%)	12 (23)
Prednisone, mg/day	5 (5–7.5)
Methotrexate, n (%)	4 (8)
Chloroquine, n (%)	3 (6)
Bosentan, n (%)	2 (4)
Sildenafile, n (%)	1 (2)

Data represent mean ± SD or median (IQR) when data were not normally distributed. Esophageal manometry assessment was available only for 14 patients. BMI: body mass index; CRP: C reactive protein; LDL: low-density lipoprotein. HDL: high-density lipoprotein, SS: systemic sclerosis. HOMA: homeostatic model assessment. NSAIDs: Non-steroidal anti-inflammatory drugs. SCORE: Systematic Coronary Risk Assessment. FVC: forced vital capacity; FEV: forced expiratory volume; DLCO: diffusion capacity of the lung for the carbon monoxide.

**Table 2:** Demographics of limited and diffuse systemic sclerosis.

	SSc Limited (n = 44)	SSc Diffuse (n = 9)	*p*
Demographics			
Female, n (%)	41 (93)	8 (89)	0.54
Age, years	59 ± 11	60 ± 5	0.81
BMI, kg/m^2^	28 ± 5	29 ± 9	0.54
Waist circumference, cm	97 ± 12	99 ± 21	0.73
Hip circumference, cm	105 ± 9	107 ± 16	0.82
Waist to hip ratio	0.93 ± 0.07	0.92 ± 0.10	0.76
Cardiovascular comorbidity			
Hypertension, n (%)	17 (39)	4 (44)	0.99
Current smoking, n (%)	2 (5)	2 (22)	0.13
Diabetes, n (%)	5 (11)	1 (11)	0.99
BMI > 30 kg/m^2^, n (%)	15 (34)	2 (22)	0.70
Statins, n (%)	16 (36)	3 (33)	0.99
Aspirin, n (%)	15 (34)	2 (22)	0.70
SCORE2 calculator			
SCORE 2, %	3.8 (2.3–6.2)	2.9 (2.7–7.3)	0.94
SCORE2 categories, n (%)			
Low to moderate	27 (61)	6 (67)	
High	14 (32)	2 (22)	0.86
Very high	2 (7)	1 (11)	
Analytical data			
Malondialdehyde, nmol/mL	1.6 ± 0.7	1.1 ± 0.4	0.098
CRP, mg/dL	1.9 (0.6–4.1)	5.2 (0.9–5.6)	0.11
Cholesterol, mg/dL	210 ± 32	206 ± 38	0.51
Triglycerides, mg/dL	202 ± 97	206 ± 41	0.28
HDL cholesterol, mg/dL	51 ± 12	61 ± 13	0.044
LDL cholesterol, mg/dL	118 ± 30	104 ± 34	0.12
LDL:HDL cholesterol ratio	2.4 ± 0.8	1.7 ± 0.6	0.008
Non-HDL cholesterol, mg/dL	159 ± 30	145 ± 32	0.20
Lipoprotein A, mg/dL	32 (8–89)	48 (34–67)	0.33
Apolipoprotein A1, mg/dL	159 ± 24	183 ± 33	0.045
Apolipoprotein B, mg/dL	110 ± 23	100 ± 31	0.29
Apo B: Apo A ratio	0.71 ± 0.21	0.55 ± 0.17	0.023
Atherogenic index	4.29 ± 1.20	3.44 ± 0.63	0.026
Insulin resistance indices			
Glucose, mg/dL	100 ± 23	102 ± 18	0.59
Insulin, µU/mL	12.6 (6.4–25.3)	10.9 (5.5–18.5)	0.57
C-peptide, ng/mL	5.3 ± 3.4	3.7 ± 1.9	0.19
HOMA2-IR	1.7 (0.9–3.4)	1.5 (0.7–2.4)	0.60
HOMA2-S%	61 (30–117)	67 (41–147)	0.60
HOMA2-B%-C-peptide	210 ± 111	153 ± 57	0.17
Systemic sclerosis related data			
Disease duration, years	9 (3–12)	9 (5–9)	0.82
Modified Rodnan Skin Score, units	4 (1–8)	6 (1–12)	0.81
Raynaud phenomenon, n (%)	39 (89)	9 (100)	0.57
Digital ulcers, n (%)	7 (16)	3 (33)	0.35
Calcinosis, n (%)	7 (16)	3 (33)	0.35
Arthritis, n (%)	3 (7)	0 (0)	0.99
Gastric reflux, n (%)	23 (52)	7 (78)	0.27
Pathological esophageal manometry, n (%)	3 (33)	4 (80)	0.27
Nailfold capillaroscopy pattern			
Early	8 (36)	1 (25)	
Active	13 (59)	2 (50)	0.47
Late	1 (5)	1 (25)	
Interstitial lung disease, n (%)	4 (10)	3 (33)	0.11
FVC, %	99 ± 14	84 ± 18	0.027
FEV1, %	101 ± 17	90 ± 17	0.076
DLCO, %	85 ± 16	75 ± 14	0.18
Pulmonary hypertension, n (%)	1 (3)	1 (14)	0.30
Autoantibodies			
Anti-centromere antibody positivity, n (%)	33 (80)	2 (25)	0.004
Anti-Scl70 antibody, n (%)	3 (7)	5 (63)	0.001
Therapies			
Current NSAIDs, n (%)	7 (16)	2 (22)	0.64
Current prednisone, n (%)	9 (20)	3 (33)	0.41
Prednisone, mg/day	5 (5.7.5)	7.5 (5–7.5)	0.55
Methotrexate, n (%)	3 (7)	1 (11)	0.54
Chloroquine, n (%)	2 (5)	1 (11)	0.44
Bosentan, n (%)	0 (0)	2 (22)	0.026
Sildenafile, n (%)	1 (2)	0 (0)	0.99

Data represent means ± SD or median (IQR) when data were not normally distributed. Esophageal manometry assessment was available only for 14 patients. BMI: body mass index; CRP: C reactive protein; LDL: low-density lipoprotein. HDL: high-density lipoprotein, SS: systemic sclerosis. HOMA: homeostatic model assessment. NSAIDs: Non-steroidal anti-inflammatory drugs. SCORE: Systematic Coronary Risk Assessment. FVC: forced vital capacity; FEV: forced expiratory volume; DLCO: diffusion capacity of the lung for the carbon monoxide.

**Table 3:** Relation of demographics and disease-related data to malondialdehyde serum levels.

	MDA, nmol/mL			
	β Coefficient (95% CI), *p*			
	Univariable		Multivariable	
Demographics				
Female	0.4 (−0.4–1)	0.35		
Age, years	−0.0007 (−0.02–0.02)	0.94		
BMI, kg/m^2^	0.003 (−0.03–0.04)	0.88		
Waist circumference, cm	−0.0003 (−0.02–0.01)	0.97		
Hip circumference, cm	−0.005 (−0.02–0.01)	0.61		
Waist to hip ratio	1 (−2–4)	0.45		
Cardiovascular comorbidity				
Hypertension	−0.06 (−0.5–0.4)	0.76		
Current smoking	−0.2 (−1–0.7)	0.62		
Diabetes	−0.2 (−0.8–0.4)	0.56		
BMI > 30 kg/m^2^	0.003 (−0.03–0.04)	0.88		
Statins	0.2 (−0.2–0.6)	0.34		
Aspirin	0.5 (0.03–0.9)	0.036		
SCORE2 calculator				
log SCORE 2	−0.04 (−0.3–0.2)	0.75		
SCORE2 categories				
Low to moderate	ref.			
High	−0.07 (−0.5–0.4)	0.75		
Very high	−0.1 (−1–0.8)	0.80		
Analytical data				
CRP, mg/dL	0.04 (−0.02–0.09)	0.16	0.03 (−0.03–0.08)	0.33
Cholesterol, mg/dL	0.006 (−0.0006–0.01)	0.077	0.006 (0.0004–0.01)	0.037
Triglycerides, mg/dL	0.00005 (−0.002–0.002)	0.96		
HDL cholesterol, mg/dL	−0.2 (−0.8–0.04)	0.50		
LDL cholesterol, mg/dL	0.008 (0.0009–0.01)	0.026	0.008 (0.001–0.01)	0.017
LDL:HDL cholesterol ratio	0.3 (0.07–0.6)	0.014	0.3 (0.04–0.5)	0.024
Non-HDL cholesterol, mg/dL	0.007 (0.0008–0.01)	0.028	0.008 (0.001–0.01)	0.017
Lipoprotein A, mg/dL	−0.005 (−0.003–0.002)	0.70		
Apolipoprotein A1, mg/dL	−0.001 (−0.009–0.006)	0.71		
Apolipoprotein B, mg/dL	0.008 (0.0002–0.02)	0.044	0.008 (0.004–0.02)	0.039
Apo B: Apo A1 ratio	0.9 (−0.06–2)	0.066	0.8 (−0.1–2)	0.085
Atherogenic index	0.2 (0.006–0.3)	0.043	0.2 (−0.01–0.3)	0.070
Insulin resistance indices				
Glucose, mg/dL	0.003 (−0.007–0.01)	0.58		
Insulin, µU/mL	−0.003 (−0.1–0.008)	0.60		
C-peptide, ng/mL	−0.02 (−0.08–0.05)	0.63		
HOMA2-IR	−0.02 (−0.1–0.07)	0.61		
HOMA2-S%	0.002 (−0.0009–0.004)	0.20		
HOMA2-B%-C-peptide	−0.001 (−0.003–0.0006)	0.18	−0.001 (−0.003–0.0007)	0.21
Systemic sclerosis related data				
SS type				
Limited	ref.			
Diffuse	−0.5 (−1–0.007)	0.053	−0.4 (−1–0.09)	0.098
Disease duration, years	0.02 (−0.01–0.05)	0.19	0.01 (−0.02–0.04)	0.52
Modified Rodnan Skin Score, units	−0.006 (−0.03–0.02)	0.64		
Raynaud phenomenon	−0.2 (−0.9–0.5)	0.56		
Digital ulcers	−0.05 (−0.6–0.5)	0.85		
Calcinosis	0.1 (−0.4–0.6)	0.71		
Arthritis	0.5 (−0.4–1)	0.24		
Gastric reflux	−0.4 (−0.8–0.005)	0.053	−0.05 (−0.09- (−0.1))	0.014
Pathological esophageal manometry	−0.5 (−1–0.4)	0.23		
Nailfold capillaroscopy pattern				
Early	ref.			
Active	0.01 (−0.5–0.5)	0.96		
Late	−0.06 (−1–0.9)	0.90		
Interstitial lung disease	−0.06 (−0.7–0.5)	0.83		
FVC, %	0.0004 (−0.01–0.01)	0.95		
FEV1, %	0.005 (−0.1–0.01)	0.93		
DLCO, %	0.01 (−0.008–0.02)	0.23		
Pulmonary hypertension	−0.02 (−1–1)	0.97		
Autoantibodies				
Anti-centromere antibody positivity	0.2 (−0.3–0.6)	0.49		
Anti-Scl70 antibody	−0.1 (−0.7–0.5)	0.71		
Therapies				
Current NSAIDs	0.2 (−0.3–0.7)	0.49		
Current prednisone	0.3 (−0.1–0.8)	0.16	0.4 (−0.1–0.8)	0.12
Prednisone, mg/day	−0.05 (−0.3–0.2)	0.64		
Methotrexate	−0.5 (−1–0.2)	0.17	−0.5 (−1–0.2)	0.17
Chloroquine	−0.4 (−1–0.4)	0.34		
Bosentan	−0.7 (−2–0.8)	0.34		
Sildenafile	1 (0.06–3)	0.041	1 (−0.2–3)	0.092

Malondialdehyde (MDA) is the dependent variable in this analysis. Multivariable analysis is adjusted for aspirin intake. Esophageal manometry assessment was available only for 14 patients. BMI: body mass index; CRP: C reactive protein; LDL: low-density lipoprotein. HDL: high-density lipoprotein, SS: systemic sclerosis. SCORE: Systematic Coronary Risk Assessment. HOMA: homeostatic model assessment. NSAIDs: Non-steroidal anti-inflammatory drugs. FVC: forced vital capacity; FEV: forced expiratory volume; DLCO: diffusion capacity of the lung for the carbon monoxide.

**Table 4:** Carotid, aorta, and echocardiogram findings in relation to MDA.

		Malondialdehyde, pg/mol			
Carotid Peak Systolic Velocity, cm/s		β Coefficient (95% CI), *p*			
Right common carotid artery	73 ± 19	−0.004 (−0.01–0.007)	0.51		
Left common carotid artery	81 ± 23	−0.003 (−0.01–0.005)	0.45		
Right internal carotid artery	72 ± 20	−0.0009 (0.01–0.01)	0.87		
Left internal carotid artery	73 ± 19	0.002 (−0.009–0.01)	0.73		
Ratio right common/internal	1.07 ± 0.35	−0.2 (−0.8–0.4)	0.46		
Ratio right common/internal ≥ 2	1 (2)	−0.7 (−2–0.7)	0.32		
Ratio left common/internal	1.16 ± 0.38	−0.2 (−0.8–0.3)	0.44		
Ratio left common/internal ≥ 2	1 (2)	−0.3 (−2–1)	0.64		
Carotid end-diastolic velocity					
Right common carotid artery	22 ± 7	0.002 (−0.03–0.03)	0.99		
Left common carotid artery	23 ± 8	−0.008 (−0.03–0.02)	0.56		
Right internal carotid artery	27 ± 10	−0.01 (−0.03–0.008)	0.24		
Left internal carotid artery	28 ± 9	−0.003 (−0.03–0.02)	0.82		
Subclinical carotid atherosclerosis					
cIMT, carotid intima media thickness, mm	0.643 ± 0.152	−0.7 (−2–0.7)	0.32		
Carotid plaque, n (%)	28 (59)	−0.07 (−0.5–0.3)	0.74		
Aorta diameter, cm	1.5 ± 0.2	0.02 (−1–1)	0.97		
Echocardiogram					
Tricuspid Annular Plane Systolic Excursion (TAPSE), mm	24 ± 4	0.08 (−0.5–0.7)	0.79		
Left ventricular ejection fraction, %	66 ± 10	−0.04 (−0.06–(−0.02))	0.001	−0.04 (−0.06–(−0.02))	0.001
Tricuspid regurgitation maximum pressure gradient (TRPG), mm/hg	20 ± 7	0.03 (−0.006–0.06)	0.099	0.01 (−0.02–0.05)	0.47
Peak velocity of the E wave of mitral filling, cm/s	79 ± 18	−0.002 (−0.01–0.01)	0.80		
Peak velocity of the A wave of mitral filling, cm/s	80 ± 19	0.001 (−0.01–0.01)	0.80		
E/a ratio	1.04 ± 0.34	−0.08 (−0.7–0.6)	0.81		
E/e’ ratio	9.9 ± 4.1	0.03 (−0.05–0.1)	0.44		
Left atrium anteroposterior diameter, mm	3.5 ± 0.5	−0.04 (−0.5–0.4)	0.84		

Malondialdehyde is the dependent variable in this analysis. E/e’ ratio: ratio of early diastolic mitral inflow velocity to early diastolic mitral annulus velocity. Adjusted for aspirin intake, and total cholesterol and apolipoprotein B serum levels.

## Data Availability

The data sets used and/or analyzed in the present study are available from the corresponding author upon request.
